# The Examination of Conscience: A Preliminary Study on the Effects on Metamemory After a 2-Week Practice

**DOI:** 10.3389/fpsyg.2022.838381

**Published:** 2022-05-04

**Authors:** Nicola De Pisapia, Martina Dall’Avanzi

**Affiliations:** Department of Psychology and Cognitive Science, University of Trento, Rovereto, Italy

**Keywords:** contemplation, mental training, memory, metacognition, contemplative science

## Abstract

Examination of conscience (EC) is a contemplative practice that consists in examining daily the actions performed during the day (evening examination, immediately before going to bed) and the actions to be performed on the coming day (morning examination, immediately after sleep). While research on contemplative practices such as mindfulness has seen a large increase of studies in recent years, research into the psychological effects related to the practice of the EC has been largely ignored in the scientific literature. On the other hand, on a careful historical and philosophical analysis, it appears evident that references to it abound in many cultural contexts and in different eras. Here, we examined the effects of a 2-week program of this practice that participants performed using a digital application developed *ad hoc* for this experiment. A control group performed an activity of the same duration, also with the support of a digital application, but which consisted of listening to audio excerpts of an Italian literary novel. Measurements taken from both groups before and after the 2-week period consisted of self-assessments of metamemory capacity, that is, awareness and knowledge of their own memory processes. Results showed that participants reported a significant decrease in two properties of their metamemory after training, specifically vividness and coherence. No other significant changes were found between the two groups. Specifically, we found no differences in other metamemory characteristics, no differences in prospective or retrospective memory, and no differences in changes in stress levels. These results, although preliminary, suggest that a relatively short period of EC does indeed make people metacognitively more aware of the limitations and errors of their memory, in particular concerning vividness and coherence.

## Introduction

In this study, we have analyzed some of the effects produced by engaging in a contemplative practice that is largely unknown in scientific research, although the practice itself is widespread in various cultures. The name of this practice is examination of conscience (EC) and it is a contemplative practice consisting of a daily review of the actions performed during the day. Usually, the practitioner performs this exercise in the evening before going to sleep (evening examination) and then immediately after waking up recapitulates the sequence of events of the past day (morning examination). During the morning examination, the practitioner additionally examines the actions he or she intends to perform the following day.

This practice has a long historical tradition worldwide, and particularly in Western and Eastern cultures. Variations of EC are described as practiced in the Pythagorean School in the 6th century BC (Huffman, 2014), in Stoicism of the Hellenistic and Ancient Roman period, for example, as described by Epictetus (Torode and Epictetus, 2017), or in the writings of the emperor Marcus Aurelius (Aurelius, 2011), in classic Tibetan Buddhist teachings (Dorje, 2004), as well as in the Christian spiritual exercises of St. Ignatius of Loyola (Loyola, 2007), just to mention a few references. For a complete historical excursus of this contemplative exercise—beyond the scope of this manuscript—see De Pisapia (2020).

As with other contemplative practices, different cultural backgrounds place emphasis on different aspects of the EC practice (in Pythagoreanism, for example, the emphasis is on memory training, while in Christianity, the purpose is to test conformity to religious law), but otherwise the procedural aspect of this exercise remains fairly constant. The essence of this exercise is still that the trainees make a daily examination in their own conscience of the actions they have performed during the day and those they will perform in the coming day. In this type of exercise, the constituent elements of the actions are as follows: (1) they should be practiced close to sleep; (2) there is a review (examination) of the salient episodes of the previous day and a contemplation of their value in terms of an ethical frame of reference (conscience) within which the individual wishes to grow and improve; and (3) there is a more conscious preparation for what the individual will do in the coming day, in accordance with the values he/she cultivates. Notice how the closeness to sleep might be a fundamental requirement, in fact in current scientific research, it is well know that sleep itself is a phase in which the experiences made during the day are consolidated, memory formation is promoted and the individual prepares for new activities (Diekelmann and Born, 2010).

This contemplative practice differs considerably from contemporary forms of mindfulness meditation (Kabat-Zinn, 2015), in which the main purpose is to direct attention to the present moment, and thus not to the past nor to the future. In particular, in one of its main exercises of mindfulness trainings, the meditation practice consists in focusing the attention on the bodily sensations linked to act of breathing. This is very different from the EC, where the focus is instead on practicing retrospective and prospective memory during the evening and morning examinations.

Given these premises, the recurrence of this practice for personal improvement in the history of Eastern and Western thought points to the centrality of this technique. It would seem, therefore, that this practice could be of enormous interest in the scholarly study of the diversity of contemplative experiences and practices. On the contrary, to our knowledge, there is currently no scientific research on the behavioral and psychological effects of this practice. Given that research on other contemplative practices is producing a large number of studies or giving possibilities of new clinical intervention for some psychopathologies, such as is happening in the field of mindfulness research (Baer, 2003), we believe that scientific investigation should also extend to other techniques belonging to contemplative traditions, but which are still ignored by current research. The purpose of the present study is therefore to conduct an initial exploratory investigation into the possible effects of the EC and to help lay the groundwork for future research on another culturally well-supported practice in the emerging field of contemplative studies. We believe that such exploration is important because it can provide preliminary methodological insights and initial results on this practice.

As a premise, we emphasize that the participants in this experiment were randomly selected. Therefore, we did not control for the particular value systems (religious, philosophical, ethical, etc.) that they might have associated with this particular practice. Rather, we simply developed a digital application consisting of audiofiles, which guided the participants to keep track of the procedural components of the exercise (evening and morning examination). Therefore, the focus of the present study was solely on the possible metacognitive effects of the exercise. Metacognition refers to the awareness and understanding of one’s cognitive processes (Flavell, 1979). In recent years, one particular view that is emerging concerning contemplative practices is that they promote metacognitive self-regulatory and modulatory capacities, enabled by the repeated exercise of introspective awareness of one’s own mental processes and behaviors (Dorjee, 2016). In the EC takes place a training of introspection in retrospective and prospective memory, therefore in this study, the specific metacognitive aspect that we scrutinized is metamemory. Such ability for metacognition of memory processes includes both the awareness and the knowledge of memory processes. The awareness component of metamemory concerns the monitoring and the regulation of memory, whereas the knowledge component corresponds to the general beliefs that a person has on his/her own memory processes (Perfect and Schwartz, 2002). Both components play a fundamental role in the recognition of our own limitations and competences in memory, for example, in the awareness and regulation of memory biases. These are particular types of cognitive biases that alter either the content or the recall of a memory, and they are very frequent both in healthy individuals, for example, concerning autobiographical memories (Romano et al., 2020), and in the development of mental disorders, such as anxiety (Coles and Heimberg, 2002) or psychosis (Eisenacher and Zink, 2017).

In this experiment, a group of participants performed the EC daily, both in the evening (retrospective) and in the morning (retrospective and prospective), using a digital application designed specifically for this study. As a control group, a homogeneous number of participants used another digital application with which they listened to an audiobook. This control group activity was performed before going to sleep and then continued after waking for a total of 2 weeks, in parallel with the experimental group. The choice of a 2-week period was dictated by the fact that training in this contemplative practice is very intensive (twice a day), and in other effective similarly intensive memory training studies, there was a similar duration (e.g., Buschkuehl et al., 2012).

The digital application consisted of a simple list of audio files to listen to before falling asleep or in the morning upon awakening. For the experimental group, the pre-recorded voice indicated to carry out the EC, namely, in the evening reviewing the salient episodes of the past day, and in the morning again to repeat the salient episodes of the day before and the main actions to be carried out on the new day. For the control group, the pre-recorded voice read brain sequentially from a novel.

Both groups completed a series of questionnaires before and after the experimental and control activities. A reliable method for measuring the subjective aspects of metamemory is to ask participants to self-assess their memory performance using questionnaires (Mäntylä et al., 2010). This method is also valid more generally for measuring metacognitive aspects, where self-assessments are useful to distinguish between subjective components of *bias* (automatic subjective inclinations), of *sensitivity* (ability to judge one’s mistakes), and *efficiency* (subjective sensitivity with respect to a certain level of task performance; Fleming and Lau, 2014). In this study, we used the Prospective and Retrospective Memory Questionnaire (Smith et al., 2000) and the Memory Experience Questionnaire (Luchetti and Sutin, 2016) that allowed us to investigate changes in metamemory after the 2-week training with the EC.

Additionally, we administered the Perceived Stress Scale (PSS; Cohen et al., 1994) to monitor possible stress-related mood changes throughout the period. Data collection took place during a lockdown in Italy to counteract the pandemic COVID-19, and all individuals who participated had already undergone at least 2 weeks of quarantine and were severely restricted in their movements. The possible conditioning effects of the lockdown on the cognitive system, induced by the stressful context, must therefore be taken into account in the overall evaluation of the results (Ingram et al., 2021). To control for cognitive effects linked to the special lockdown conditions, we had a control group that run in parallel to the experimental group.

Additionally, given our hypothesis that EC acts on metamemory (both retrospectively and prospectively), we asked our experimental and control participants after the 2-week period whether they had experienced a change in their ability to be lucid during dreaming. The parallel that we draw between EC and lucid dreaming is only theoretical and exploratory, and motivated as follows. A lucid dream is a dream in which the dreamer is aware that he/she is dreaming. It is an infrequent mental phenomenon, in which the experiences during a dream are correctly recognized by the dreamer as of a dream type thanks to an increased self-awareness. The lucid dreamer knows that he/she is within a dream context and in a virtual world made of corporeality, sensations, people, and stories that he/she experiences in full awareness of their hallucinatory character. This recognition is accompanied by an integral memory of one’s waking life, and sometimes by an ability to guide one’s actions with self-awareness (clarity) and proactivity (intentionality). Metacognitive (Filevich et al., 2015) and more specifically metamemory skills (Holzinger and Mayer, 2020) seem to be central in this mental phenomenon (Baird et al., 2019; De Pisapia, 2021). Furthermore, the EC is practiced close to sleep, and several techniques to increase the occurrence of lucid dreaming similarly involve retrospective and prospective metamemory training, for example, the so-called Mnemonic Induction of Lucid Dream, MILD (La Berge, 1980).

## Materials and Methods

### Participants

A total of 44 participants took part in this research. We chose to determine the sample size based on comparatively similar experimental psychology studies on memory training, such as, for example, Richmond et al. (2011). They were recruited online (email and advertisement on social networks), and all contacts with them throughout the experiment took place remotely. The excluding criteria were the presence of neurological or psychological disorders (as self-reported by the participants, and not determined through an external assessment). Participants were randomly assigned to the two experimental groups, 22 in the control group and 22 in the experimental group. Three individuals from the experimental group did not complete the questionnaires, and consequently, they were excluded from the study. The average age was 35.75 years, with a SD of 7.2. The control group consisted of 7 males and 15 females (age *M* = 31.8, *SD* = 7.0), while the experimental group consisted of five males and 14 females (age *M* = 40,3, *SD* = 6.7).

Over a 2-week period, participants in both groups were required to use a digital application and listen to a few short audio files at two moments during the day, namely, in the evening before going to bed and in the morning after waking up. Before listening to the audio guide, participants were asked to find a quiet place where they could not be disturbed. They were then asked about the location of the exercise and when they expected to go to bed (for the evening practice) or how long it had been since they had woken up (for the morning practice). These questions allowed us to verify that participants actually performed the exercises when they were instructed to do so, i.e., just before going to bed and after waking up. After this brief questionnaire, participants began listening to the audio files included in the application.

Participants in the experimental group then listened to the instructions for the EC exercise, which were of course different in the morning and in the evening. These instructions followed the simple indications on the practice that can be found in the contemplative texts mentioned in the Introduction. They simply consisted of asking the participants to recall the main sequence of episodes from the day (evening exercise) and to both recapitulate the main sequence of events from the previous day and mentally repeat the main sequence of actions they planned to take during the day (morning exercise; see Supplementary Material for the full transcript of the instructions).

Participants in the control group instead listened to audio excerpts from a book of Italian literature in the evening and in the morning sessions (Zeno’s Conscience by Italo Svevo). This is a classic novel of Italian literature written in the first person as a kind of therapeutic diary of the protagonist describing some of the most important episodes of his life. We chose the reading of the literary novel because (1) it was an actual activity (thus, the control group was not passive or in a waiting list); (2) it engaged the control group for a time comparable to the EC practice (both in the evening and in the morning); (3) it was not a contemplative practice in itself; and (4) it did not affect metamemory directly.

A series of self-assessment tests were administered online to participants in both groups before (pre) and after (post) the entire 2-week program. The effects measured were related to the experience of memory, their perception of prospective and retrospective memory, stress, and the tendency to develop lucidity in dreams. Below is a description of the self-assessment measures.

### Measures

#### Memory Experiences Questionnaire

The memory experiences questionnaire (MEQ) in the short version (Luchetti and Sutin, 2016) is a memory self-assessment test consisting of 34 questions, with answers given on a five-point Likert scale ranging from totally disagree to totally agree. There are 10 subscales that measure the phenomenological qualities of autobiographical memories: Vividness, Coherence, Accessibility, Sensory details, Emotional intensity, Visual perspective, Time perspective, Sharing, Distancing, and Valence. Each scale of the abbreviated form has a good internal consistency, a high correlation with the extended version (Sutin and Robins, 2007).

#### Prospective Memory and Retrospective Memory

The Prospective and Retrospective Memory Questionnaire (PMRQ; Smith et al., 2000) is a self-assessment questionnaire of memory failures in the form of 16 questions (e.g., “Do you fail to recall things that have happened to you in the last few days?”). Answers are given on a five-point Likert scale from 1 corresponding to “never” to 5 corresponding to “very often,” where an increase in the scoring corresponds to a perception of a worsening of memory. There is a global scoring, but results are also divided into the prospective and retrospective memory subscales, which investigate the two memories separately. Notice how a scoring increase in this questionnaire corresponds to an increase in the perceptions of memory failures.

#### Perceived Stress Scale

The Perceived Stress Scale (PSS) (Cohen et al., 1994) is a test for assessing the perception of stress. The questionnaire consists of 10 sentences, and for each of them, the participant must evaluate how stressful the situations described are by giving a score on a five-point Likert scale ranging from 0 = never to 4 = very often.

#### Lucid Dreaming

At the end of the 2-week period using the digital application, we asked every participant of both groups to answer this simple question: “In the last 2 weeks have you noticed an increase in the awareness of dreaming while dreaming?.” The answers could vary on a scale from 1 to 5 (1-Totally disagree; 2-Partially disagree; 3-Neither agree nor disagree; 4-Partially agree; and 5-Totally agree).

### Data Analysis

The responses on each questionnaire were scored according to their protocols, which resulted in one score per participant and time point for each of the scale or subscale. The distribution of scores on all the dependent variables was evaluated prior to conducting primary analyses; because the data were not normally distributed, we used permutation tests, which are non-parametric tests as they do not rely on assumptions about the distribution of the data and can be used with different types of scales and with a small sample size.

For each experimental and control participant, we computed gain scores for each measure by subtracting the pretest scores from the posttest scores and then running independent sample *t*-test on gain score differences between the groups. We used permutational *t*-tests with the “pairwise.perm.t.test” function from the “RVAideMemoire” package in R (by Hervé and Hervé, 2020). The difference between the traditional and the permutational *t*-tests is that while the traditional *t*-test determines the equality of the group mean, whereas the permutation version tests the exchangeability of the group observations. The number of permutation was set to 10,000. We applied the false discovery rate (FDR) correction method (Benjamini and Hochberg, 1995) to account for Type I errors introduced by multiple pairwise tests and Type II errors introduced by small sample size, setting statistical significance at *p* = 0.05.

## Results

Full results are summarized in Table 1. In particular, results from the *t*-tests of the gain scores in the MEQ showed differences in two subscales between the two groups, showing how the experimental group decreased the values in the self-assessment of two of the subscales. In particular, the subscale of Vividness of the memory decreased significantly in the experimental group (gain score *M* = −0.611, *SD* = 2.593) compared to the increase of controls (gain score *M* = 1.476, *SD* = 3.586) with a mean difference between the two groups of *M* = −2.087 (value of *p* = 0.047, FDR corrected). Furthermore, the subscale Coherence between the remembered events decreased significantly in the experimental group (gain score *M* = −2.111, *SD* = 2.988) compared to a slight decrease of controls (gain score *M* = −0.238, *SD* = 1.998) with a mean difference between the two groups of *M* = −1.873 (value of *p* = 0.027, FDR corrected).

**Table 1:** Gain scores (means M and SD) of metamemory measures, stress, and lucid dreaming self-assessments in the experimental group and the control group, and then, their differences tested with *t*-test permutation (false discovery rate, FDR corrected).

Variable	Experimental group		Control group		Experimental vs. control		
	Gain scores		Gain scores		Gain differences		
	*M*	*SD*	*M*	*SD*	Mean diff	*t*-value	*p*-value
MEQ	3.833	10.596	0.524	7.507	3.309	0.404	0.280
Vividness	**-0.611**	**2.593**	**1.476**	**3.586**	**-2.087**	**−1.775**	**0.047**
Coherence	**−2.111**	**2.988**	**−0.238**	**1.998**	**−1.873**	**−5.949**	**0.027**
Accessibility	0.333	4.339	0.190	3.140	0.143	−2.021	0.930
Sensorial details	1.111	4.739	1.381	4.914	−0.270	−0.267	0.890
Emotional intensity	−0.278	4.638	−0.381	3.956	0.103	−2.634	0.970
Visual perspective	0.611	2.789	−0.238	3.659	0.849	−2.466	0.440
Temporal perspective	0.000	3.308	0.333	2.331	−0.333	−2.824	0.760
Sharing	−0.944	4.151	0.000	4.572	−0.944	−2.705	0.550
Distancing	0.889	2.988	0.429	2.271	0.460	−1.934	0.640
Valence	2.056	8.680	−2.048	6.771	4.104	−1.277	0.110
PMRQ	0.941	4.723	−1.050	3.410	1.991	−2.331	0.160
Prospective memory	−0.059	2.727	−0.650	2.739	0.591	−4.053	0.550
Retrospective memory	1.000	3.518	−0.400	1.847	1.400	−2.607	0.150
PSS	−2.000	6.872	−1.000	6.221	−1.000	−2.874	0.650
Lucid dreaming	**3.200**	**1.146**	**2.333**	**1.175**	**0.867**	**5.238**	**0.047**

*Statistically significant gain score differences are in bold*.

Results of the PMRQ showed instead no significant differences between the two groups (experimental vs. control group, gain score *M* = 1.991, *SD* = −2.331, value of *p* = 0.16). The perception of prospective memory remained fundamentally unchanged in both groups (gain scores *M* = −0.059 and *SD* = 2.727 in the experimental group, gain scores *M* = −0.65 and *SD* = 2.739 in controls). Notably, the retrospective memory was generally perceived in the experimental group as worsened from pre to post (gain scores *M* = 1.000 and *SD* = 3.518 in the experimental group, *M* = −0.400 and *SD* = 1.847 in controls) with a total perception of retrospective memory change of *M* = 1.4, even though this difference was not significant (*p* = 0.150, FDR corrected).

As for the stress, in the PSS, we found no significant differences between the two groups in the pre vs. post-comparison (experimental vs. control group, gain score *M* = −1.0, *SD* = −2.874, value of *p* = 0.65).

Finally, concerning the self-reported occurrence of lucid dreams, the experimental group reported a significant increase in the occurrence of lucidity and awareness in dreams (gain score *M* = 0.867, value of *p* = 0.047, FDR corrected) with scores of *M* = 3.200 and *SD* = 1.146 for the experimental group and *M* = 2.333 and *SD* = 1.175 for the control group.

## Discussion

In this study, we examined the effects of a 2-week program consisting of evening and morning exercises of EC supported by a digital application. A control group performed an activity of the same duration, also with the support of a digital application, but consisting of listening to audio excerpts of an Italian literary novel. Measurements conducted on both groups before and after the 2-week period showed that the experimental group participants’ self-assessment of some properties of their metamemory decreased. Specifically, we noted a significant decrease in Vividness, which refers to the visual clarity and visual intensity of the recalled memories, and Coherence, which refers to the extent to which the memory involves a logical story in a particular time and place rather than just fragments of the original experience. Another change we noted was a significant increase in participants’ self-assessed capacity to develop lucidity in their dreams. No other significant changes were found between the two groups. In particular, we found no differences in other properties of metamemory in general, no differences in prospective or retrospective memory, and no differences in changes in stress levels.

While it may seem counterintuitive that people who undergo metamemory training become worse at metamemory, one possible explanation is that this exercise makes people more aware of the limitations and fallacies of their memory. Metamemory consists of awareness and knowledge of our own memory processes (Perfect and Schwartz, 2002), and the actual familiarity and exploration of our beliefs about our own memory processes triggered by the EC exercise had the effect in our 2-week protocol of making participants more aware of their limitations and memory errors. The metamemory properties that decrease are specifically Vividness and Coherence of memories. For these measures, participants made a self-assessment of the ability to see their memory clearly and to reconstruct the sequence of remembered events, and to check whether they appeared in memory vividly and in the form of a logical and coherent story. Vividness refers to the visual clarity and visual intensity of retrieved memories, and it has been described as the most important aspect of autobiographical memory (Greenberg and Rubin, 2003). Coherence refers instead to the extent to which a retrieved memory involves a logical story in a specific place and time rather than experiential fragments (Sutin and Robins, 2007). These subscales are therefore of central importance and very instructive for the practice of EC, where the exercise consists mainly in carrying out a daily screening of actions performed and yet to be performed, with the result that participants became much more aware of what their mind was actually capable of performing when they tried to vividly and logically reconstruct their memory or intentions.

As for the significant improvement in lucid dreaming, the extreme limitation of the method used (a simple self-assessment question) certainly does not allow a definite conclusion. However, this datum points to the need to further explore the possibility that metamemory trainings such as EC may indeed elicit greater metacognitive capacity during dreaming, even in the context of a more severe assessment of one’s memory abilities.

This experiment represents only a preliminary attempt to investigate EC, a contemplative practice that belongs to a wide variety of traditions. Apart from the interest of these preliminary results and the method used (the use of a digital application and the comparison with an active control group), the study has a number of limitations. One of these relates to the fact that the experiment was conducted during the COVID-19 lockdown period, which severely restricted the movements and diversity of experiences of participants who were forced to work or study from home. This may have influenced the outcome in ways that are difficult to assess. Another limitation is that we did not measure the level of religiosity or spiritual orientation of the participants, in order to ensure that experimental and control groups were comparable on these measures. Another limitation is the duration of the protocol of only 2 weeks, which leaves open the question of what would happen if this practice was longer and more stable. Another limitation is that the participants were not assessed or excluded based on conditions that might potentially impact the results, such as, for example, sleep disorders or substance use/medication. Finally, the questionnaires used were exclusively self-assessments, so that only subjective aspect of both metamemory and lucidity in dreaming was measured and no conclusions could be drawn about objective memory performance or verified frequency change in lucid dream occurrence.

In conclusion, despite these significant limitations, all of which can be addressed in future studies, we believe this study is important because it provides some methodological insights and initial interesting results on this contemplative practice, which has otherwise been ignored in the scientific literature despite being widely used throughout human history and in various cultures around the world.

## Data Availability Statement

The datasets presented in this article are not readily available because the participants in the study only gave permission to the researchers to use their data. Requests to access the datasets should be directed to nicola.depisapia@unitn.it.

## Ethics Statement

The studies involving human participants were reviewed and approved by Research Ethics Committee University of Trento. The patients/participants provided their written informed consent to participate in this study.

## Author Contributions

NP and MA designed the study. MA programmed the digital application, collected the data, and performed the analysis. NP lead in writing the manuscript and supervised the project. All authors contributed to the article and approved the submitted version.

## Conflict of Interest

The authors declare that the research was conducted in the absence of any commercial or financial relationships that could be construed as a potential conflict of interest.

## Publisher’s Note

All claims expressed in this article are solely those of the authors and do not necessarily represent those of their affiliated organizations, or those of the publisher, the editors and the reviewers. Any product that may be evaluated in this article, or claim that may be made by its manufacturer, is not guaranteed or endorsed by the publisher.
