# Supplementary material for: The Examination of Conscience: A Preliminary Study on the Effects on Metamemory After a 2-Week Practice
Source: Front Psychol. 2022 May 4;13:838381. doi: 10.3389/fpsyg.2022.838381 (PMC9114734; doi:10.3389/fpsyg.2022.838381)
Supplement: Supplementary file 1 [file Data_Sheet_1.PDF]

SUPPLEMENTARY INFORMATION

The examination of conscience: a preliminary study on the effects on metamemory after a two-week practice

Nicola De Pisapia, Martina Dall'Avanzi

Text of the audio guide for the examination of conscience

First evening
1. Well let's get started. Find a comfortable position, relax, and if you prefer, close your eyes.
Think back to the things done and happened today, starting from the moment of awakening. Try to look back on the day in your mind as if it were a movie or video. Try to retrace the main things that happened and your actions starting from the first thing, then the one immediately after and so on throughout the day up to this moment. Pay attention to the temporal succession of events.
2. Good. You're done for tonight. Good night.

First morning
1. Well let's get started. Find a comfortable position, relax, and if you prefer, close your eyes.
Quickly think back to the things you did yesterday, in the order in which they happened and how you rethought them before falling asleep. Try to remember the salient moments and actions.
2. Well, now think about the main things you are going to do today. Think about the first thing you will do, the second and so on. Try to build a mental schedule with the succession of all the things you will do, until you get to the evening and the time you go to bed. Try to create this mental list by paying attention to the temporal succession of the actions you will do.
3. Now that you have thought about what you are going to do today, have a nice day

Second and subsequent evening
1. Well let's get started. Find a comfortable position, relax, and if you prefer, close your eyes.
Think back to the things done and happened today, starting from the moment of awakening. Try to look back on the day in your mind as if it were a movie or video. Try to retrace the main things that happened and your actions starting from the first thing, then the one immediately after and so on throughout the day up to this moment. Pay attention to the temporal succession of events.
Quickly review the things that happened and done yesterday and in the following days.
2. Now that you have rethought everything you did today and yesterday, try to rethink what you set out to do this morning, think again and evaluate how things went.
3. Good. You're done for tonight, good night.

Second and subsequent morning
1. Well let's get started. Find a comfortable position, relax, and if you prefer, close your eyes.
Quickly think back to the things you did yesterday, in the order in which they happened. Quickly think back to some of the things that happened the previous day, try to remember the salient moments and actions.
2. Well now think about the main things you will do today. Think of the first thing you will do as soon as you get up, the second, and so on. Try to build a mental schedule with the succession of all the things you will do

until you get to the evening and the time you go to bed. Try to create this mental list by paying attention to the temporal succession of the actions you will do.

3. Now that you have thought about what you are going to do today, have a nice day.
